# Drivers of Crimean-Congo Hemorrhagic Fever in Natural Host and Effects of Control Measures, Bulgaria

**DOI:** 10.3201/eid3109.241952

**Published:** 2025-09

**Authors:** Georgina Limon, Simona R. Tchakarova, Anna Ludi, Tsviatko Alexandrov, Iva Christova, Petya Petkova, Emmanuel Maze, Kelly Thomas, Natalie Baker, Marion England, Clare Browning, Ginette Wilsden, Sandra Belij-Rammerstorfer, Teresa Lambe, Anna Jolles, Miles Carroll, Roger Hewson, Simon Gubbins, Bryan Charleston, Nicholas A. Lyons

**Affiliations:** The Pirbright Institute, Pirbright, UK (G. Limon, A. Ludi, E. Maze, M. England, C. Browning, G. Wilsden, S. Gubbins, B. Charleston, N.A. Lyons); Bulgarian Food Safety Agency, Sofia, Bulgaria (S.R. Tchakarova, T. Alexandrov, P. Petkova); National Center of Infectious and Parasitic Diseases, Sofia (I. Christova); UK Health Security Agency Porton Down, Salisbury, UK (K. Thomas, N. Baker, R. Hewson); University of Oxford, Oxford, UK (S. Belij-Rammerstorfer, T. Lambe, M. Carroll); Pandemic Science Institute, Oxford (T. Lambe, M. Carroll); Oregon State University, Corvallis, Oregon, USA (A. Jolles); London School of Hygiene and Tropical Medicine, London, UK (R. Hewson)

**Keywords:** Crimean-Congo hemorrhagic fever, Crimean-Congo hemorrhagic fever virus, CCHFV, vector-borne infections, viruses, zoonoses, exposure, force of infection, vaccine efficacy, spatial-temporal, control measures, Bulgaria

## Abstract

Crimean-Congo hemorrhagic fever (CCHF) is an emerging tickborne disease and a World Health Organization priority. Although humans are accidental hosts, infection can lead to hemorrhagic fever with a high fatality rate. Domestic animals play a critical role in disease transmission, but infected animals do not show clinical signs and viremia is short; thus, CCHF virus (CCHFV) infections can remain unobserved. During 2017–2019, we conducted 2 sequential observational studies followed by a multisite randomized controlled trial to determine spatial-temporal patterns and quantify drivers for CCHFV exposure in a natural host (sheep) in a CCHF-endemic area of Bulgaria. We found high-risk areas embedded in endemic regions. Animal characteristics were not correlated with seropositivity; however, a seasonality effect was observed, suggesting sampling time was a potential confounder. Force of infection varied across farms and over time. CCHFV transmission heterogeneity among farms is driven by preventive measures used to reduce exposure to ticks.

World Health Organization–designated priority emerging diseases are those with potential to cause severe epidemics without available or sufficient medical countermeasures (*1*). Crimean-Congo hemorrhagic fever (CCHF), a severe tickborne zoonotic disease with a high fatality rate in humans (*2*), is a priority emerging disease. Fatality rates have gradually increased in recent decades; major differences exist across geographic regions and occupations (*3*). The etiologic agent, CCHF virus (CCHFV), has a wide geographic distribution and is endemic in parts of Africa, Asia, Eastern Europe, and the Middle East. CCHFV is transmitted by ticks belonging to the Ixodidae family, mainly of the genus *Hyalomma* (*2*,*4*). Rising environmental temperatures influence CCHFV transmission to new geographic areas, in conjunction with other factors, such as travel, trade, livestock movement, and wild bird migration (*5*).

Animals, including domestic livestock, can become infected when bitten by CCHFV-infected ticks. Although animals develop a transient viremia, they do not exhibit clinical signs (*6*–*8*). Small ruminants have been suggested as good proxies to monitor the presence of CCHFV in a given region (*6*,*9*). Sheep have been epidemiologically linked to human exposure to the virus and cases (*10*–*12*). Despite many serologic studies being conducted in different settings, using various tests and a wide range of study designs (*6*,*13*–*15*), very little is known about the factors driving differences in CCHFV infection, host humoral responses, and spatial patterns of exposure in livestock. We conducted 2 sequential observational studies followed by a multisite randomized trial to determine spatial patterns and main drivers for CCHFV exposure in sheep (a natural host) in Bulgaria. Written consent was obtained from all participating sheep farmers. Ethics approvals were obtained from the Bulgarian Food Safety Agency ethics committee and The Pirbright Institute’s Animal Welfare Ethical Review Board.

## Methods

### Study Design

**Figure 1:**
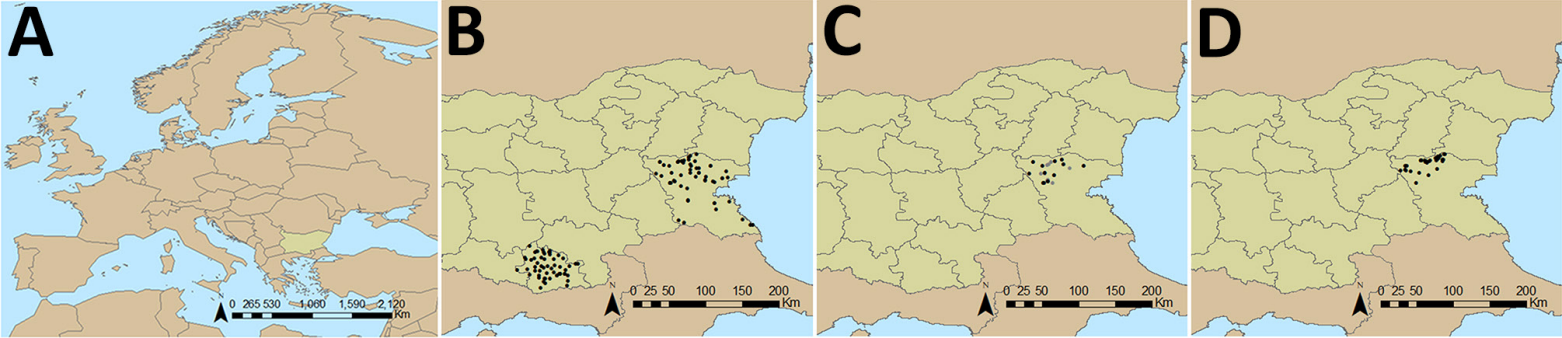
Geographic locations of sheep farms in study of drivers of Crimean-Congo hemorrhagic fever in natural host and effects of control measures, Bulgaria. A) Location of Bulgaria within Europe. B) Location of sheep farms that were part of cross-sectional field study 1 (n = 120). Black dots indicate sheep farms in Kardzhali Province, located in the southern part of Bulgaria, and Burgas Province, located in the southeastern part of the country. C) Location of sheep farms that were part of the follow-up field study 2 during March 2018 in Burgas Province (n = 25). Black dots indicate farms that were part of both field studies 1 and 2 (n = 14). D) Location of farms that were part of the multisite randomized control trial (field study 3) in Burgas Province (n = 32). Black dots indicate sheep farm locations.

We conducted a cross-sectional study (field study 1) in October 2017 in which we recruited 120 commercial sheep farms in the CCHFV-endemic provinces of Burgas and Kardzhali, Bulgaria, after the main tick-biting season. In Europe, temperature and photoperiod are key drivers of tick seasonality; the optimum environmental temperature for tick activity is 20°C–25°C. In the Balkans, those conditions occur during March–October (*16*). We calculated the target sample size for each province to estimate the proportion of seropositive sheep with 95% confidence and 6% precision for an expected seroprevalence of 50% and 0.12 intrafarm correlation (Appendix). We selected 5 lambs (3–12 months of age) and 5 sheep (13–24 months of age) within each participating farm and collected blood samples from each selected animal. We collected animal and farm level data by using a standardized questionnaire in a mobile application (Appendix Table 1, Figure 1).

We conducted a follow-up observational study (field study 2) before the next tick-biting season (before March 2018) within the main hotspot area (northwestern part of Burgas province), identified in the cross-sectional study, to investigate the potential effects of age and seasonality on seropositivity in sheep. We visited 25 farms; 14 of those had been included during the first field study. We sampled 15 sheep at each farm, stratified by age: 5 lambs (<12 months), 5 young adults (13–36 months), and 5 adults (>36 months). We collected blood samples from each selected animal and corresponding animal data (age, sex, breed, presence of ticks).

We then conducted a multisite, randomized, 2-arm, triple-blinded, controlled trial (field study 3) in the previously identified hotspot to determine the efficacy of a modified vaccinia virus Ankara (MVA)–vectored vaccine candidate (encoding the CCHFV envelope spike glycoprotein [GP]) in sheep during periods of expected high transmission levels (i.e., natural challenge of animals) and to estimate the force of infection (FOI) over time and across farms. In addition, we used the placebo group to better elucidate profiles for CCHFV nucleoprotein (NP) and GP Gc IgGs over time after natural exposure. The vaccine candidate has been shown to be immunogenic and 100% protective in mice (*17*) but poorly immunogenic in sheep; only a modest increase in CCHFV Gc IgG has been observed in sheep under controlled conditions (*18*). We calculated sample size by considering an incidence of 16.9% in the unvaccinated group, a between cluster variation of 0.021, and 20 animals per farm and by assuming a vaccine efficacy of 50%, 95% confidence, and 80% power (Appendix). We also assumed a 15% loss to follow-up would occur during 6 months.

We recruited 32 commercial sheep farms into field study 3. At each of those farms, we selected 20 lambs that were 2–4 months of age and already weaned. Lambs were the unit of randomization, and we allocated them in equal numbers to either the vaccine group (arm 1) or placebo (received phosphate-buffered saline) group (arm 2). We administered a vaccine booster 4 weeks after the primary dose. We conducted intention-to-treat analysis; seroconversion was the endpoint. We visited each farm and collected blood samples at 2, 4, 10, 13, 17, 21, and 27 weeks after primary dose. We gathered animal data and general information on farm characteristics, such as management practices and biosecurity, during the first visit by using an electronic standardized questionnaire. During each follow-up visit, we collected data on changes and preventive measures (deworming, tick control, and shed spraying for vector control) that had been administered between visits. We used the EpiCollect5 tool (https://five.epicollect.net) to collect the data for all studies. 

### Sample Storage and Laboratory Methods

We stored all field study serum samples in duplicate aliquots at −20°C. We shipped 1 aliquot per sample at the end of each field study to The Pirbright Institute (Pirbright, UK) for testing. We tested serum samples in duplicate by using an in-house indirect ELISA to detect CCHFV antigen-specific IgG responses (CCHFV NP and Gc IgGs), as previously described (*18*). We also tested serum samples from CCHFV-seropositive animals in the controlled trial for CCHFV RNA by real-time reverse transcription PCR at the Bulgarian Food Safety Agency laboratories, as previously described (*19*). We tested samples from when the animals first seroconverted and from the previous sampling period before the sheep seroconverted.

### Statistical Analysis

For field study 1, we estimated weighted sheep seroprevalence. To assess the level of clustering of seropositive sheep within farms, we estimated the intrafarm correlation coefficient for seropositive status of individual sheep by using the farm variance from a mixed effect model, considering farm as a random effect. We conducted risk factor analysis separately at both the animal and farm level. 

At the animal level, we tested the extent to which animal characteristics were associated with individual serostatus (outcome variable) by using mixed effect models, including farm as a random effect. We assessed collinearity between all predictor variables for which p was <0.1 in the univariate analysis and, when collinearity was present (Pearson correlation >0.8), we kept only 1 variable in the model. We generated multivariable models by using a backward stepwise selection procedure with likelihood ratio tests to compare models with and without the variable of interest.

**Figure 2:**
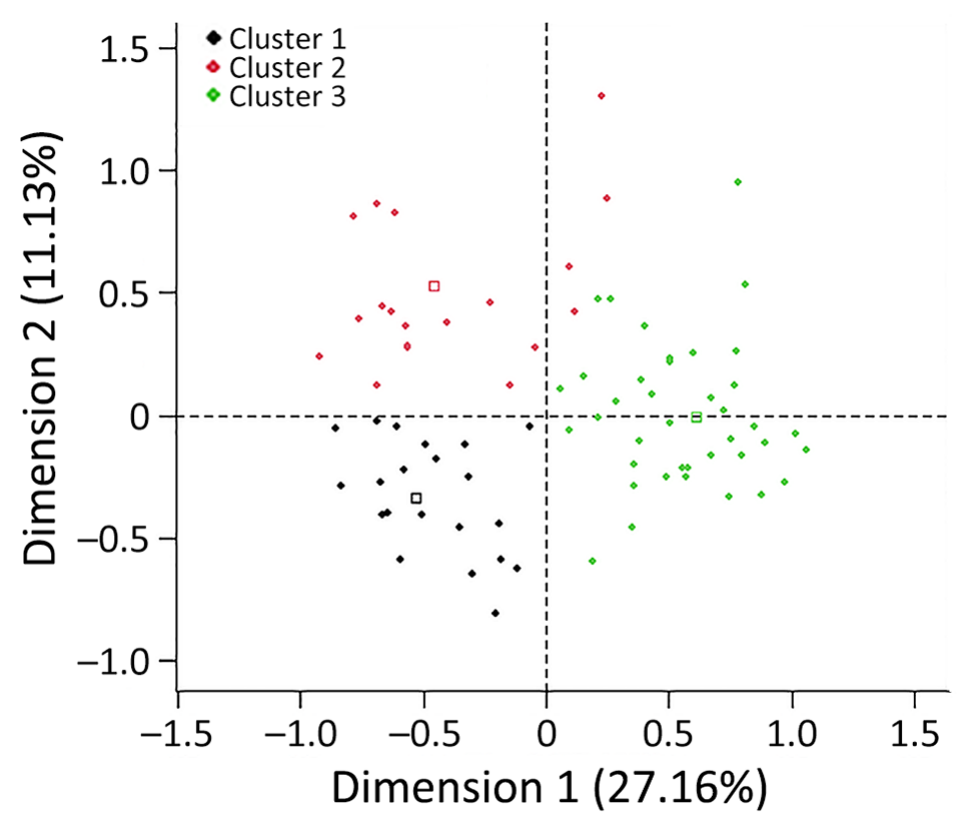
Farm typologies identified after hierarchical cluster analysis in study of drivers of Crimean-Congo hemorrhagic fever in natural host and effects of control measures, Bulgaria. First, multiple correspondent analysis was performed to transform correlated variables into a small number of synthetic uncorrelated factors. Hierarchical cluster analysis was then used to group farms into clusters according to their level of similarity with respect to the factors created by the multiple correspondent analysis. Data were collected during October 2017 from 120 commercial sheep farms in Burgas and Kardhzali Provinces in Bulgaria. Typology (cluster) 1 (n = 54) comprised mixed farms, most of which were located in Kardhzali Province. Lambs were kept outdoors during the day and indoors at night, and all farms reported applying tick control by spraying animals with acaracides. Typology (cluster) 2 (n = 26) comprised most of the meat farms located in either Kardhzali or Burgas Province. Most farms kept lambs outdoors during the day and indoors at night. One third of those farms did not use tick control measures for animals. Typology (cluster) 3 (n = 40) comprised most of the dairy farms; most were located in Burgas Province. Lambs were kept indoors at all times until weaning. Most farms reported applying tick control either by dipping or spraying animals with acaracides.

At the farm level, we recategorized farm and management practices after data exploration (Appendix Table 1). We used data reduction techniques to identify farm typologies on the basis of management practices and farm characteristics (Appendix Tables 2, 3, Figure 2). To assess the extent to which environmental factors affect the risk for CCHFV exposure of sheep on a farm, we used land cover (shrub, cultivated, or arboreal), mean normalized difference vegetation index (NDVI), and NDVI spring slope (*20*) as proxies to capture environmental traits that shape the distribution of *Hyalomma* spp. mosquito activity and seasonal dynamics. We tested the extent to which farm typologies and environmental variables were associated with the number of seropositive animals in the farm by using Poisson regression; we used the number of animals sampled as an offset. We selected final multivariable models by using a backward selection process with 1 variable removed each time. We then used a likelihood ratio test to assess which model best fit the data.

We generated choropleth maps of empirical Bayes smoothed rate at the municipality level to explore potential spatial clustering of CCHFV seropositive animals. We explored spatial autocorrelation of the smoothed Bayes risk at a global scale by using the Moran’s *I* statistic and at a local scale by using the Getis-Ord GI* statistic.

For field study 2, we only considered farms that were visited during both field studies 1 and 2 (n = 14) and animals having the same age range (3–24 months) to capture farm-level dynamics of CCHFV NP and Gc seropositivity at key seasonal timepoints (October 2017 and March 2018). We used multivariable mixed effect models to assess differences in seropositive animals between the 2 sampling periods and age groups, including farm as a random effect.

For field study 3, we determined the number of unvaccinated and vaccinated lambs seroconverting during each sampling period for each farm. We assumed a lamb seroconverted during the first sampling period when its CCHFV NP IgG status changed from negative to positive. We used the pattern of seroconversion to estimate FOI for each lamb (Appendix Figure 3). FOI varied among farms and among sampling periods and incorporated the effects of control measures (vaccination, deworming, spraying, and tick control), enabling their efficacy to be quantified. In particular, vaccine efficacy was calculated by the formula 1-λ_1_/λ_0_, where λ_1_ is the FOI in vaccinated lambs and λ_0_ is the FOI in unvaccinated lambs (*21*).

We used descriptive statistics to characterize CCHFV NP and GP Gc profiles in the placebo groups. We estimated median, interquartile range, and fold-change relative to day 0 for each sampling point. We used Spearman correlation coefficients (ρ) and 95% CIs to determine the relationship between CCHFV NP and Gc IgGs.

## Results

To explore the potential effect of seasonality, we assessed the difference in the number of seropositive animals between sampling periods (October 2017 and March 2018) considering only farms that were visited on both field studies 1 and 2 (n = 14) and animals from the same age groups (3–24 months). Sampled animals were not the same in both studies but came from the same farms; therefore, we expected the same management practices and levels of exposure. All farms were dairy farms, all but 1 farm reported deworming their animals regularly, and all but 1 farm reported performing regular tick control. On the 14 farms, 55/140 (39.3%) sheep were CCHFV Gc IgG seropositive in October 2017 and 22/58 (37.9%) sheep were seropositive in March 2018, whereas 75/140 (53.6%) sheep were CCHFV NP IgG seropositive in October and 5/58 (8.6%) sheep were NP IgG seropositive in March. We observed a strong seasonality effect (p<0.001) after adjusting for age group. Sheep were more likely to be NP IgG seropositive at the end of the tick biting period (October sampling), when CCHFV transmission is expected to be higher, than at the end of the winter (March sampling), when CCHFV transmission is expected to be low (Table 1; Appendix Tables 4, 5). However, we did not observe a seasonality effect for CCHFV Gc antibody levels, suggesting that CCHFV NP IgG might be a better marker of recent exposure than CCHFV Gc IgG. Therefore, we considered CCHFV NP IgG levels to be a main indicator of recent natural exposure for the cross-sectional and controlled trial studies.

**Table 1:** Results from multivariable mixed-effect models used to assess serologic associations in study of Crimean-Congo hemorrhagic fever in sheep and effects of control measures, Bulgaria*

Characteristic	CCHFV glycoprotein Gc IgG positive			CCHFV nucleoprotein IgG positive	
	aOR (95% CI)	p value		aOR (95% CI)	p value
Study type					
Follow-up, March 2018, n = 58	Referent			Referent	
Cross-sectional, Oct 2017, n = 140	1.31 (0.64–2.75)	0.464		14.49 (5.54–46.93)	<0.001
Age category					
13–24 mo., young adult sheep, n = 115	Referent			Referent	
3–12 mo., lambs, n = 83	1.44 (0.74–2.82)	0.283		1.8 (0.94–3.68)	0.073

*Associations between CCHFV glycoprotein Gc or nucleoprotein seropositivity and time of sampling (study type) and age; only farms visited during both field studies 1 and 2 (n = 14) and sheep of the same age (3–24 mo) (total no. = 198) were considered. Models had farm as a random effect. Results from univariate analysis are in the Appendix Tables 1, 2. aOR, adjusted odds ratio; CCHFV, Crimean-Congo hemorrhagic fever virus.

We included 120 farms and 1,200 sheep in the cross-sectional study (Figure 1). The overall weighted NP IgG seroprevalence was 38.5% (95% CI 35.3%–42.0%); we observed a higher seroprevalence in Burgas Province than in Kardzhali Province (Appendix Table 6). Dairy breeds were more likely to be seropositive than mixed breeds. The province and breed type exhibited strong collinearity; most dairy breeds came from Burgas Province, where most dairy farms were located and where univariate models were used. We did not find statistically significant associations between serologic status and sex, age, or presence of ticks at the time of sampling (Appendix Table 7). At the farm level, 105 (87.5%) farms had >1 seropositive sheep. Adjusting for land cover, farms from typology (cluster) 3 were more likely to have seropositive animals than farms in typology 2 (Figure 2; Appendix Table 8). Farms within 5 km of cultivated or arboreal land cover were more likely to have seropositive animals (Appendix Table 8).

**Figure 3:**
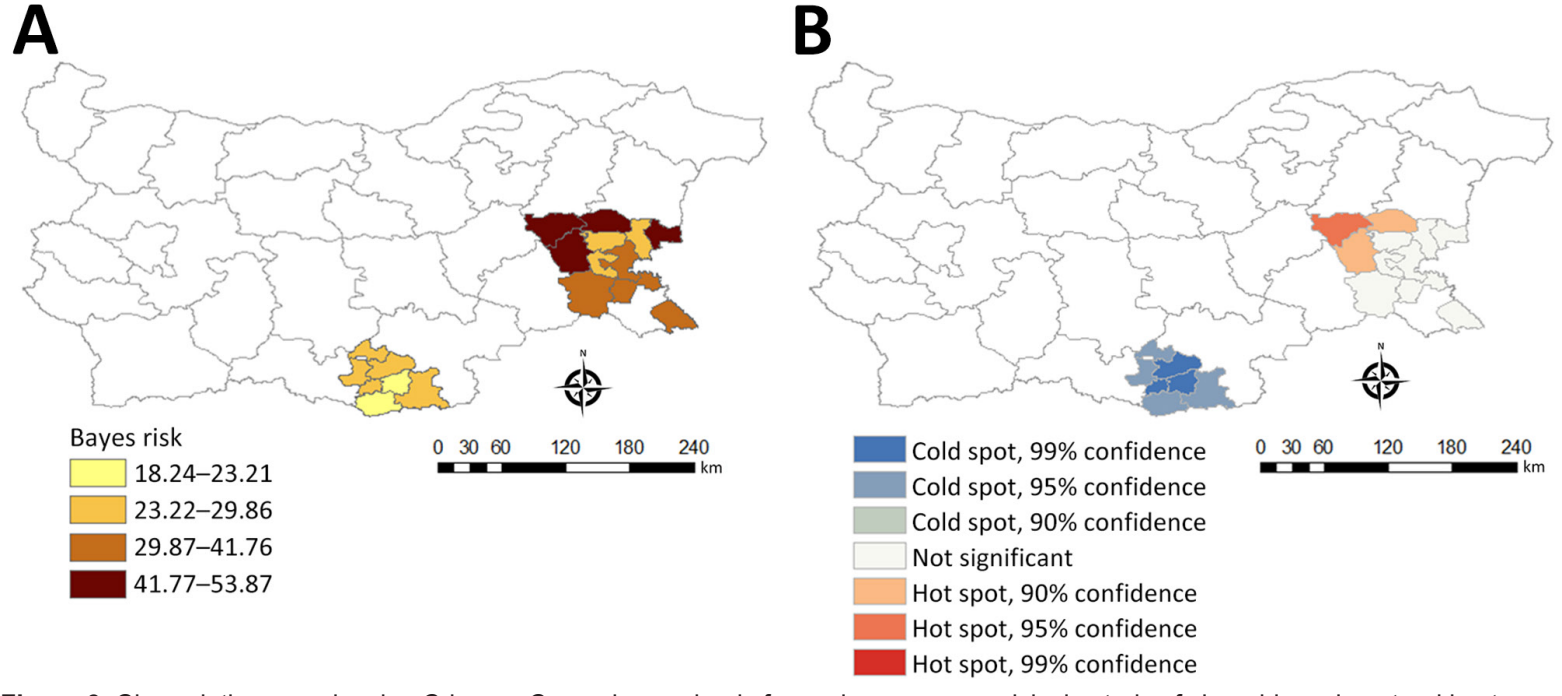
Choropleth maps showing Crimean-Congo hemorrhagic fever virus exposure risks in study of virus drivers in natural host and effects of control measures, Bulgaria. Bayes smoothed rate (A) and Getis-Ord Gi* hotspots (B) of Crimean-Congo hemorrhagic fever virus nucleoprotein Ig in sheep are indicated for municipalities in Burgas Province (southeastern part of the country) and Kardzhali Province (southern part of country). Data were collected during October 2017.

We found CCHFV seropositivity throughout both provinces studied; however, we found spatial heterogeneity at farm and municipality levels. Some level of clustering of seropositive animals occurred within farms; the overall intrafarm correlation was 0.25. Bayes smoothed rates of seropositive animals varied across municipalities. We identified higher risk for CCHFV exposure in northwestern Burgas (Figure 3). We observed a significant positive spatial autocorrelation at the municipality level (Moran’s *I* = 0.36; p = 0.001), indicating nearby observations were more similar on average than distant ones.

The multisite randomized controlled trial comprised 32 commercial sheep farms and 640 lambs (20 per farm) (Appendix Table 9), which we followed up for 6 months. In the placebo group (n = 320), CCHFV Gc IgG was detectable at baseline but declined early during the study, consistent with waning passive immunity, whereas NP IgG levels rose during June–September, aligning with the expected peak in CCHFV transmission caused by tick activity and supporting NP IgG as a marker for recent CCHFV infection (Appendix Figure 4, panels A, B). Moreover, correlation between NP and Gc IgG responses (Spearman ρ = 0.57) was moderate at the first timepoint in early March, then declined rapidly and remained weak or inconsistent thereafter (Spearman ρ range 0.25–0.41) (Appendix Figure 4, panels C, D), indicating that CCHFV NP and Gc responses are not tightly coupled, especially over time.

**Figure 4:**
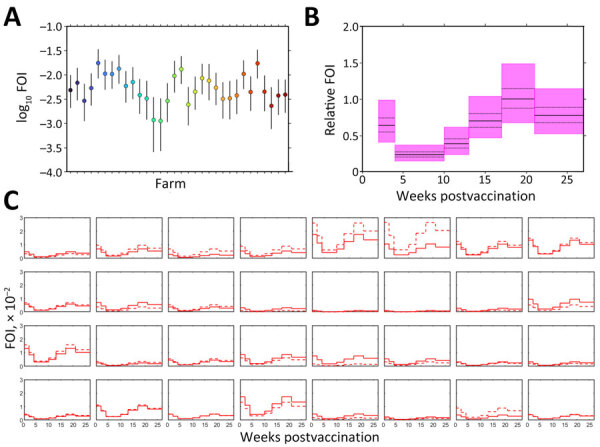
Estimated FOI of Crimean-Congo hemorrhagic fever virus for 32 commercial sheep farms in Burgas Province, Bulgaria. Sheep were vaccinated and tested for virus IgG over a 6-month follow-up. A) Baseline FOI for each farm. Circles indicate posterior medians; error bars indicate 95% credible intervals. B) Relative FOIs during each sampling period. Posterior median (solid black lines), interquartile range (dashed black lines), and 95% credible interval (purple shading) are indicated. C) Posterior median FOI for unvaccinated (solid lines) and vaccinated (dashed lines) animals in each farm. Each plot represents data from 1 farm. Seroconversion was determined on the basis of virus nucleoprotein IgG levels. FOI, force of infection.

**Figure 5:**
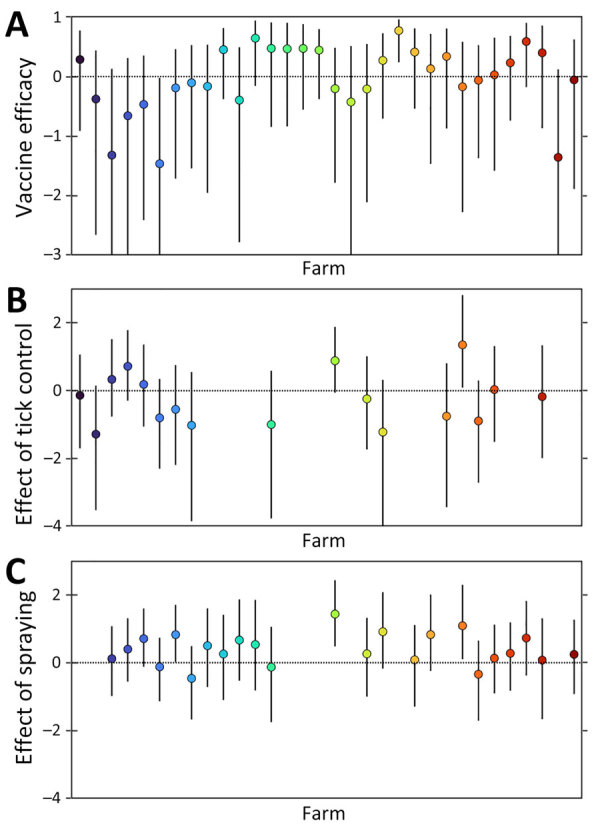
Effects of control measures on the force of infection for Crimean-Congo hemorrhagic fever virus at 32 sheep farms in Burgas Province, Bulgaria. A) Estimated vaccine efficacy of the Crimean-Congo hemorrhagic fever–modified vaccinia virus Ankara vaccine. B) Effect of tick control at farms. C) Effect of spraying. Circles indicate posterior medians; error bars indicate 95% credible intervals. Horizontal dotted line indicates no effect.

FOI varied among farms and over time (Figure 4; Appendix Table 10, Figure 5). FOI was initially high (especially during weeks 2–4), declined to a minimum during weeks 4–10, then rose again and peaked during weeks 17–21. Only 2 animals tested positive for CCHFV RNA by reverse transcription PCR; both animals were from the same farm and tested positive during the sampling period at the beginning of July (week 17). Both animals were seronegative at the previous sampling timepoint (week 13), and although ELISA optical density values for both sheep increased at the following sampling timepoint (week 27), only 1 of the sheep became seropositive.

Vaccination had a limited effect on FOI at most farms; vaccine efficacy varied among farms (Figure 5; Appendix Table 10). The posterior median vaccine efficacy was >0 (median 42.7% [range 2.8%–77.1%]) for 16 farms, although the 95% lower credible limit was >0 for only 1 farm (Figure 5).

Deworming had no apparent effect on FOI (Appendix Table 11). However, tick control in animals or spraying sheds with acaricide had an effect that varied among farms. Tick control reduced FOI (i.e., posterior median effect <0) in 11 of 17 farms (Figure 5). Spraying reduced FOI in 4 of 23 farms. The magnitude of the effect was typically larger for tick control than for spraying (Figure 5).

## Discussion

We determined epidemiologic and driver profiles for CCHFV exposure in natural hosts in a virus-endemic setting over time. Furthermore, we quantified the potential effect of different control measures on CCHFV FOI. We found spatial heterogeneity of CCHFV seropositivity at both farm and municipality levels and identified hotspots in virus-endemic areas, consistent with results in other endemic regions (*15*,*22*,*23*). In addition, we observed a critical effect of seasonality; sheep were more likely to be CCHFV NP IgG seropositive toward the end of the tick biting period, when CCHFV transmission is expected to be highest, than at the end of the winter, suggesting NP IgG is a better marker for recent CCHFV infection. Contrary to reports in previous studies (*23**–**27*), animal characteristics were not significantly associated with seropositivity. However, age was marginally associated with CCHFV NP IgG when adjusting for time of sampling, and lambs were more likely to be seropositive than were adult sheep.

Apart from nosocomial transmission, close contact with animals, farming activities, slaughtering animals, and a history of tick bites have all been reported as the main activities that can increase risks for human infection and clinical cases (*28*–*32*). In a parallel study, we took blood samples from farmers in a subset of farms that were part of the first field study (Appendix Table 12). Only 3 (6.8%) farmers were positive for CCHFV IgG. Low levels of seropositivity are to be expected in vectorborne disease–endemic areas where humans are accidental hosts. The proportion of seropositive farmers was slightly higher in this study than that previously reported within the general population in Bulgaria (2.8%) and Greece (4.2%) (*28*,*33*). Two of the 3 seropositive farmers lived in the high-risk area identified in northwestern Burgas Province, suggesting that CCHFV exposure is an occupational risk and not only derived from individual risk activities.

To better elucidate the dynamics of the humoral response toward CCHFV over time, we quantified temporal variation in FOI on the basis of antibody responses over a 6-month period. FOI was initially high, which might be a consequence of maternal antibodies found in lambs that were part of the study. FOI then rose again during the summer period (weeks 17–21 of the study), likely because of the higher tick activity during those months leading to increased CCHFV transmission. CCHFV NP has been shown to be highly immunogenic; NP antibodies are produced during infection in humans and mice (*34*,*35*). Further studies should be conducted to formally assess the protective role of maternal CCHFV antibodies.

FOI has been suggested to influence vaccine efficacy (*36*). Our findings indicate that FOI and vaccine efficacy varied across farms, and vaccination had a limited effect on FOI in this setting (*18*). In contrast, tick control and spraying reduced FOI, suggesting that CCHFV transmission heterogeneity among farms in high-risk areas is driven by different farm management practices and preventive measures used to reduce tick exposure. Further studies should be conducted to assess differences in tick density at the farm and animal level.

Seroepidemiologic studies have contributed to delineating transmission dynamics for various vectorborne zoonotic diseases (*37*–*40*). Our findings indicate that, given the short period of infectivity, serologic analysis is more reliable than other methods to assess CCHF dynamics in domestic animals.

In conclusion, we provide insight into the epidemiology and drivers of CCHFV transmission. As with most tickborne diseases, CCHFV dynamics are complex. We have identified key epidemiologic parameters derived from empirical data in a natural host species, in CCHFV-endemic settings, and over time. Because of the limitations in conducting controlled challenge studies with category 4 pathogens and the lack of a robust correlation of protection, natural challenge studies are a reliable approach to evaluate the efficacy of vaccine candidates and other control measures.

Appendix Additional information for drivers of Crimean-Congo hemorrhagic fever in natural host and effects of control measures, Bulgaria.
