# Supplementary material for: Drivers of Crimean-Congo Hemorrhagic Fever in Natural Host and Effects of Control Measures, Bulgaria
Source: Emerg Infect Dis. 2025 Sep;31(9):1738–46. doi: 10.3201/eid3109.241952 (PMC12407213; doi:10.3201/eid3109.241952)
Supplement: Appendix — Additional information for drivers of Crimean-Congo hemorrhagic fever in natural host and effects of control measures, Bulgaria. [file 24-1952-Techapp-s1.pdf]

Article DOI: https://doi.org/10.3201/eid3109.241952

EID cannot ensure accessibility for supplementary materials supplied by authors. Readers who have difficulty accessing supplementary content should contact the authors for assistance.

Drivers of Crimean-Congo Hemorrhagic Fever in Natural Host and Effects of Control Measures, Bulgaria

Appendix

Material and Methods

Field Study 1: Cross-Sectional Study

Study Design

A cross-sectional study was conducted in the provinces of Burgas and Kardzhali. Those provinces were selected according to previous reports of high levels of Crimean-Congo hemorrhagic fever virus (CCHFV) infection in livestock and humans (*1–4*). The provinces also represent different agroecological characteristics and sheep production systems. The target sample size for each province was calculated to estimate the proportion of seropositive sheep with 95% confidence and 6% precision for an expected seroprevalence of 50%. This is considered the worst-case scenario and was chosen given the lack of accurate estimates of prevalence in the study area. To account for the potential clustering of sheep within farms and the multistage level of sampling, the resulting number of animals (n = 257) was adjusted for the intracluster (intrafarm) correlation coefficient (ICC). In the absence of estimates of ICC for CCHF in sheep (and livestock in general), we used the median ICC from published estimates of other tickborne diseases, which was 0.12 (*5*,*6*). Therefore, 60 farms in each region and 10 animals per farm (n = 600 animals per province) were sampled to assess sheep seroprevalence with the desired precision and level of confidence.

Samples and Data Collection

Sixty farms with >20 sheep were randomly selected in each province by using the official records of sheep flocks, provided by the Bulgarian Food Safety Agency (BFSA), as a sample

frame. In addition, 10 reserve farms were selected in each province to be used if farmers from the selected farms refused to take part in the study or did not have enough eligible animals to be sampled. Five lambs (3–12 months old) and 5 sheep (13–24 months) were systematically selected in each selected farm. For this, eligible animals were selected as they passed through the door of the pen using a herd size specific sampling interval. The sampling interval wa estimated by dividing the number of lambs (or 13–24 sheep) within the farm by 5. A minimum age of 3 months was chosen to avoid potential false positives caused by maternal antibodies, whereas the upper limit (24 months) was chosen to avoid the effect of exposure over the years. We assumed that animals aged 3 to 12 months would have been through 1 peak tick activity season, whereas sheep 13 to 24 months old would have lived through 2 peak tick activity seasons. Up to 9 mL of blood was collected from the jugular vein of each selected animal by a qualified veterinarian using prelabeled vacutainer tubes. Date of sampling, age, sex, breed, and on-host presence of ticks at time of sampling were recorded. In addition, farm coordinates and information on farm characteristics, management practices, and farmers knowledge of CCHF and potential risk behaviors were gathered using a standardized questionnaire.

In a parallel study, at each selected farm, 2 persons in charge of sheep management and in close contact with the animals on a day-to-day basis were asked to be sero-sampled. From those who accepted, a visit was subsequently arranged for a qualified technician to visit the farm and collect blood samples. Date of sampling, age, sex, main duties in the farm, history of tick bite, and history of vaccination were recorded.

Farm, animal, and farmer data were collected using a mobile application for data collection (https://five.epicollect.net). Each farm was given a unique study identification number to link blood samples, animal, and farmer data to the farm of origin.

The animal component of this study received ethical approval from the Pirbright Institute Animal Welfare and Ethical Review Board and the Bulgarian Food Safety Agency ethical board. The human sampling component received ethical approval from the National Center of Infectious and Parasitic Diseases, Bulgaria. All farmers who took part in the study provided informed consent. Participants were informed that participation was voluntary.

Serology

Sheep blood samples were centrifuged to separate serum from blood cells at the National Diagnostic Research Veterinary Institute in Sofia. Serum samples were heat inactivated at 56°C for 30 min and stored at −20°C until they were shipped on dry ice to The Pirbright Institute, UK, where testing took place. Samples were tested in duplicate using an in-house indirect ELISA for the detection of CCHFV antigen-specific IgG responses (CCHFV-NP IgG and CCHFV-Gc IgG), as previously described (*7*). Briefly, all samples were diluted 1:125 in Blocker Casein (Thermo Scientific 37528) before testing. To ensure minimal plate to plate variation, a negative lamb serum (GIBCO/Life Technologies, Cat no. 16070–096) and positive sheep serum were included on each plate. The positive sheep field serum was identified through initial ELISA screening, as there are no commercially available CCHFV-positive serum controls.

In short, 96-well microtitration plates were coated with 1µg/mL of CCHFV antigen (NP, The Native Antigen Company, REC31639; Gc, The Native Antigen Company, REC1615) diluted in PBS. The plates were then incubated overnight at 4°C in a humidity chamber. The next day, after flicking the plates, Blocker Casein (Thermo Scientific 37528) was added to each well, and the plates were incubated for at least 2 hours at room temperature. The plates were then washed 4 times with PBS/0.05% Tween (PBS/T). The test samples and controls were added next before the plates were again incubated overnight at 4°C. The plates were washed as before and donkey anti-sheep/goat HRP-conjugated antibody (Bio-Rad Cat. STAR88P) was added at a dilution of 1:30,000 in PBS/T. The plates were again incubated at room temperature for 1 hour before being washed as before. TMB was added and 1 M sulfuric acid was used to stop the reaction. The OD of each well was read at 450 nm. Serum samples were deemed positive with an OD value of $\geq$0.234 for CCHFV-Gc and $\geq$0.225 for CCHFV-NP IgG, previously defined by applying a finite mixture model using the Expectration Maximization algorithm (*7*).

Farmers' blood samples were tested for IgG against CCHFV using a commercial ELISA test (Vector Best® Novosibirsk, Russia), which is whole-virus specific. Sample processing and testing were conducted at the National Center of Infectious and Parasitic Diseases in Bulgaria. The OD readings for the samples were used to calculate the sample positivity coefficient (PC) as described by the manufacturer. A sample with a PC value of $\geq$1 was considered positive, a value <0.8 was negative, and a value between 0.8 and 1.0 was inconclusive. Samples from farmers

positive for IgG were tested for CCHFV-specific IgM antibodies using a commercial ELISA (Vector Best® Novosibirsk, Russia).

Data Analysis

The weighted sheep seroprevalence was estimated to account for the variation in flock sizes between farms. Weighted seroprevalence was also calculated stratifying by province. As a measure of precision, 95% confidence intervals were obtained for each prevalence estimate. Intrafarm correlation (ICC) for seropositive status of individual sheep was estimated using the farm variance from a mixed effect model considering farm as a random effect.

Risk Factors at Animal Level

Descriptive statistics were obtained at animal and farmer levels. The extent to which animal and farmer characteristics (predictor variables) were associated with individual serostatus (outcome variable) was tested using mixed effect models, including farm as a random effect. Animal serostatus was determined on the basis of CCHFV-NP IgG.

Following univariate analysis of persons, collinearity was assessed between all predictor variables for which $p \leq 0.1$ in the univariate analysis and when present (Pearson correlation ≥ 0.8) only 1 variable was kept in the model. Multivariable models were generated using a backward stepwise selection procedure using likelihood ratio tests to compare models with and without the variable of interest. All models had farm as a random effect.

Farm Classification and Risk Factors at Farm Level

Descriptive statistics were obtained at farm level for variables that described knowledge of CCHF and risk behaviors that could lead to exposure to CCHFV. Farm and management practices were recategorized after data exploration (Appendix Table 1). Data reduction techniques were used to identify farm typologies on the basis of management practices and farm characteristics. First, multiple correspondent analysis (MCA) was performed to transform correlated variables into a small number of synthetic uncorrelated factors. Hierarchical cluster analysis was then used to group farms into clusters according to their level of similarity with respect to the factors created by the MCA. The first 2 components were retained, accounting for 38.3% of the variance. Province, production type, and place were lambs are kept until weaning were the characteristics that contributed the most to dimension 1, whereas production type, place

where rams come from, and type of tick control where the characteristics that contributed the most to dimension 2 (Appendix Table 3).

To assess the extent that environmental factors change the risk of sheep on a farm being exposed to CCHFV, land cover (a parcel-based thematic classification of satellite image data), mean normalized difference vegetation index (NDVI), and NDVI spring slope (*8*) were used as proxies to capture environmental traits that shape the distribution of *Hyaloma spp* activity and seasonal dynamics. NDVI mean and spring slope raster layers were downloaded from Dryad (*8*) and values were extracted for each study farm in ESRI ArcGIS using the farm coordinates collected during the fieldwork. Land cover raster layer was downloaded from Diva-GIS (*9*), a 5 km radius buffer was created around each farm to take into account the areas where animals are taken during the day to pasture, and the majority land cover category was calculated for each buffer zone. A buffer of radius of 5 km was chosen according to local vets and farmer estimates of daily animal movements.

The extent to which farm typologies identified with and environmental variables (land cover and NDVI slope) were associated with the number of seropositive animals in the farm was tested using Poisson regression, with number of animals sampled as an offset. Final multivariable models were selected using a backward selection process with 1 variable removed each time. A likelihood-ratio test was then used to assess which model best fit the data.

Spatial Analysis

Choropleth maps of empirical Bayes smoothed rate were generated at municipality level to explore potential spatial clustering of seropositive animals (*10*) using Equations 1 to 3 as follows: given that y_i equalled the number of seropositive sheep observed in the ith municipality, n_i the total number of sheep sampled in the ith municipality, r_i was the proportion of positive sheep for the ith municipality, then the pooled rate across all municipalities (γ) was calculated as:

$$\hat{y} = \frac{\sum y_i}{\sum n_i} \quad \text{(Equation 1)}$$

and the estimate of the population variance of the rate on the basis of a weighted sample of the observed rates (φ) was calculated as:

$$\varphi = \frac{\sum n_i (r_i - \gamma)^2}{\sum n_i} - \frac{\gamma}{\bar{n}} \text{ (Equation 2)}$$

then θ, the empirical Bayes-smoothed rate for the ith municipality, was calculated as:

$$\theta = \frac{\varphi * (r_i - \gamma)}{\varphi + \frac{\gamma}{n_i}} + \gamma \text{ (Equation 3)}$$

Spatial autocorrelation of the smoothed Bayes risk was explored at a global scale using the Moran *I* statistic and at a local scale using the Getis-Ord GI* statistic. The global Moran *I* statistic was used to assess the presence, strength, and direction of spatial autocorrelation over the whole study area, using a queen's contiguity weight matrix and 499 random permutations. A p-value of $\leq$0.05 was considered significant. The GI* statistic was used to detect clustering of municipalities with similar risk for CCHF and to identify the locations of hotspots or coldspots at the municipality level. The GI* statistic returned a z-score for each municipality and, for statistically significant positive z-scores, the larger the z-score the more intense the clustering of high values (hotspot). For statistically significant negative z-scores, the smaller the z- score the more intense the clustering of low values (coldspot).

Statistical analyses were performed in R 4.0.2 (R Development Core Team 2017) (*11*) using packages car (*12*), FactoMineR (*13*), lme4 (*14*), and lmtest (*15*). Spatial analyses were conducted using R 4.0.2 and tools provided in ArcGIS 10.8.1 (*16*).

Field Study 2: Follow-Up Study

A follow-up study was conducted in March 2018 to assess in more detail the potential effect of animal's age and seasonality for detection of CCHFV-NP IgG and CCHFV-Gc IgG.

Study Design

Twenty-five farms located in the hotspot area (northwest Burgas) identified in the first field study (described above) were visited during the first week of March 2018. Of the 25 farms, 14 farms had been visited during the first field work study, were still operating and had enough animals to be sampled in each category, while 11 were visited for the first time.

Fifteen sheep were sampled at each farm and stratified by age: 5 lambs (up to 12 months), five young adults (13–36 months), and five adults (>36 months). We included older

animals this time which would have lived through multiple high-tick activity seasons, potentially being exposed several times. Blood samples were collected from the jugular vein of each sheep included in the study using prelabeled vacutainer tubes. Animal data (age, sex, breed, presence of ticks) were collected for each animal sampled. Blood samples were processed and tested as in the cross-sectional study.

Data Analysis

The extent to which animal age was associated with serostatus was tested using mixed effect models including farm as a random effect. Animal ages were grouped, reflecting the sampling design (≤12 months, 13–36 months, and >36 months).

Seasonality

To assess the potential effect of seasonality, the difference in the number of seropositive animals between sampling periods (October 2017 and March 2018) was assessed considering only farms that were visited on both field study dates (n = 14) and animals of the same age (3–24 months). Multivariable mixed effect models were used to assess differences in seropositive animals between the 2 sampling periods and age groups, including farm as a random effect. Models for each of the 2 definitions of seropositivity were run separately. Statistical analyses were performed in R 4.0.2 (R Development Core Team 2017) (*11*), using packages car (*12*), lme4 (*14*), and lmtest (*15*).

Field Study 3: Multisite Randomized 2-Arm Control Trial

Study Design and Intervention Details

A multisite randomized 2-arm control trial was conducted in the northwestern part of Burgas province to (i) determine the vaccine efficacy of a modified vaccinia virus Ankara (MVA) vectored vaccine candidate encoding envelope GP spikes of CCHFV in sheep within a high-risk area and during periods of expected high levels of transmission (i.e., natural challenge of animals), and (ii) estimate the force of infection over time and across commercial sheep farms. The study area was selected according to a spatial clustering identified as part of the cross-sectional study described above.

Considering an incidence of 16.9% in the unvaccinated group, a between cluster variation (k) of 0.021, 20 animals per farm, and assuming a vaccine efficacy of 50%, a sample size of 26

farms in total and 520 animals (260 animals per group) would be required at a 95% significant level and 80% power, using the following equation 4,

$$c = 1 + \left(Z_{\alpha/2} + Z_{\beta}\right)^2 \frac{\pi_0 (1 - \frac{\pi_0}{m} + \frac{\pi_1 (1-\pi_1)}{m} + k^2(\pi_0^2 + \pi_1^2)}{(\pi_0 - \pi_1)^2} \quad (4)$$

where n is the number of animals without adjusting for between farm variation; c is the required number of sites (farms); $Z_{\alpha/2}$ is the power, Z_{β} is the confidence; π_1 and π_0 are the true proportions in the presence and absence of the intervention, respectively; m is the number of sheep from each farm and k is the between cluster coefficient of variation.

Parameters for sample size calculation (incidence and between farm variation) were determined according to results from the 2 previous observational field studies (October 2017 and March 2018) described in this publication. Given differences on incidence and between farm variation obtained in both field studies, we estimated the sample size by using different parameters derived from each field study. There was no information available on vaccine efficacy in sheep. The only study available was in mice, which reported 100% protection (*17*). We considered 3 conservative estimates: 40%, 50% and 60%. According to flock sizes in the study area and previous discussions with field vets and farmers, including 20 animals from each farm was considered a realistic number in terms of logistics and commitment expected from the farmers during the trial. The decision of which sample size to use out of the different combinations considered was decided taking into account logistics and feasibility for the follow-up component. We assumed a 15% loss in follow-up during 6 months, increasing the sample size to 598 animals, we increased to 640 animals to have equal number of animals per farm, giving a total number of 32 farms which was believed to be an achievable task.

Thirty-two sheep farms were recruited to the study as follows. Using an updated official list of farms keeping sheep in the 3 municipalities in the hotspot area, farms with at least 100 sheep were identified (n = 202). At least one eligible farmer per village was contacted and invited to participate in one of the three workshops hold to recruit farmers. During the workshop, the aim and protocol of the study was explained and questions farmers might have with regard to the trial were answered. At the end of the workshop, farmers were given a consent form (in Bulgarian) to take home, in which the aim and main points of the protocol were explained. Ten

days after the workshops, farmers were contacted again, and those who agreed were asked to provide the signed consent form.

In each farm taking part in the study, 20 lambs were selected. Lambs between 2 and 4 months old and already weaned were eligible. Selected lambs were clinically examined by a qualified vet. Lambs were the unit of randomization and were allocated to either vaccine (arm 1) or placebo (PBS) (arm 2) with the same number of animals in each arm. Vials containing the vaccine and placebo were identical and only identified by four-digit serial number previously generated in R (R Development Core Team 2017) (*11*). The list identifying each vial was kept by the project manager and an independent researcher at The Pirbright Institute. Each vial contained enough vaccine (or placebo) to vaccinate 5 animals (in the same farm) with 30% extra capacity per vial. Packs with four vials (two vials with vaccine and two vials with placebo) were prepare for each farm taking part of the study. Farmers, vets applying treatments, and persons performing the lab analysis and initial statistical analysis were blinded to the identity of the groups (triple blinded).

Prior to the administration of the vaccine (or placebo), each animal (one at the time) had two ear tags applied with a unique number and blood samples collected from the jugular vein. Animal data (ear-tag number, breed, sex, and date of birth) and vial number (from the treatment received) were recorded using an electronic form (https://five.epicollect.net).

Treatment (vaccine or placebo) was administered via a single intramuscular injection into the right hind leg of each animal using a low deadspace luer-lock syringe (2 mL syringe) and needle size of 22G (one per animal). MVA vaccine contained 10^8 plaque forming units (pfu) of MVA-GP diluted in endotoxin-free phosphate-buffered saline (PBS). MVA strain 1974/NIH clone 1 is a pox-vectored vaccine encoding CCHFV GP. A vaccine booster was administered 4 weeks after the primary dose. Intention to treat was used for further analysis, and seroconversion was the endpoint.

All lambs were observed for 30 minutes after the application of the vaccine (or placebo) for side effects and any abnormality was registered. Following 30 min of observation, animals were moved with the rest of the animals in the flock, and farmers were instructed to raise the sheep according to their usual practices. Therefore, it was expected that farmers would see animals included in the study every day.

Vials had the same serial numbers. Packs were prepared for each farm with the corresponding vials. Field teams receive a hard copy of the animals' IDs and vial number administered to match numbers during the booster and keep the study blinded. The number in the vial administered during the booster stage was registered in the electronic recording form.

Animals were followed up for 6 months. Each field team (composed of two qualified veterinarians) followed up the same farms that were assigned at the beginning of the study. Each farm was visited 2, 4, 10, 13, 17, 21 and 27 weeks after vaccination. Samplings dates were adjusted to avoid national holidays or festivities, when farmers and vets were unlikely to be available. During each visit, blood samples were collected from the jugular vein of each lamb included in the study using prelabeled vacutainer tubes. The maximum pain and distress caused to lambs was mild. Animal allocated treatment (vaccine or placebo) was disclosed to the farmers and field teams at the end of the study. Animals that received the vaccine could not enter the food chain and therefore, were culled at the end of the study via overdose of anesthetic.

Blood samples were processed and stored as in the cross-sectional study (described above). Samples were tested, in duplicate, using the same in-house indirect ELISAs than that in the observational studies. All serum samples were tested for CCHFV-Gc and CCHFV-NP IgG.

General information on farm characteristics (management practices and biosecurity) were gathered during the first visit using an electronic standardized questionnaire (https://five.epicollect.net). Information on any changes and preventive medicine (deworming, tick control, and shed spraying for vector control) administered between visits were collected in each follow-up visit.

The study received ethical approval from the Bulgarian food safety agency ethical committee and the Animal Welfare Ethical Review Board at the Pirbright Institute. The maximum pain and distress caused to lambs taking part of this study was mild.

Data Analysis of Force of Infection and Effect of Control Measures

The force of infection, $\lambda_j(t)$, for lamb j at time t is given by

$$\log \lambda_j(t) = a_{F_j} + b(t) + c_{F_j} V_j + d_{F_j} C_j(t),$$

where a_F is the baseline for farm F, $b(t)$ is the time-varying component of the force of infection (see below), c_F is the effect of vaccination for farm F and V is a variable indicating whether ($V =$

1) or not ($V = 0$) a lamb was vaccinated, d_F is the effect of the control measure (either deworming, tick control, or spraying) on farm F and $C(t)$ is a variable indicating whether (*1*) or not (0) the control measure was implemented for lamb j at time t (i.e., on the farm where it was kept). Vaccine efficacy is given by V_E = 1-exp(c_F) (*18*). Data on control measures only recorded whether the measure was used during a sampling period, so $C_j(t)$ was set to the same value (0 or 1) for the whole sampling period.

Two models were considered for the time varying component of the force of infection. In the first, the force of infection was assumed to be constant (i.e., $b(t) = 0$). In the second, a piece-wise function was used for the time-varying component of the force of infection, such that

$$b(t) = \begin{cases} 0 & 0 < t < t_1 \\ b_k & t_k < t < t_{k+1} \end{cases}$$

where t_k is the time the kth sample was taken on the farms. Here, the b_ks gives the force of infection during the kth sampling period, relative to the first.

Two models were considered for vaccine efficacy. In the first, the vaccine efficacy was assumed to be common to all farms (i.e., $c_F = c$). In the second, the vaccine efficacy varied among farms, such that it was drawn from a higher-order normal distribution (i.e., c_F~Normal(μ_c,σ_c^2)). As with vaccination, two models were considered for the effect of control. In the first, the effect was assumed to be common to all farms (i.e., $d_F = d$). In the second, the effect varied among farms, such that it was drawn from a higher-order normal distribution (i.e., d_F~Normal(μ_d,σ_d^2)). Because it was used on only six farms, the model in which the effect of deworming varied among farms did not converge and so was not considered further.

Parameters were estimated in a Bayesian framework. The likelihood for the data are given by

$$L = \prod_{j \in N} \exp\left(-\sum_{t=0}^{\min(t_{end};t_{dead}^{(j)})} \lambda_j(t) \right) \times \prod_{j \in S} \exp\left(-\sum_{t=0}^{t_{s_j-1}} \lambda_j(t) \right) \times \left(1 - \exp\left(-\sum_{t=t_{s_j-1}}^{t_{s_j}} \lambda_j(t) \right) \right),$$

where N and S are lists of lambs which remained seronegative throughout the study and which seroconverted during the study, respectively. Here, t_{end} is the time of the last sampling, t_{dead} is the

time at which a lamb died (if it died during the study period), t_s is the time at which sample s was taken, and s is the sampling at which a lamb was first seropositive.

In the models where a parameter (a, c or d) varied among farms, a hierarchical prior was used so that the parameter for each farm was drawn from a higher-order normal distribution with mean μ_j and standard deviation σ_j ($j = a,c$, or d). A normal prior with mean 0 and standard deviation 10 was used for each hierarchical mean (μ_j) and an exponential prior with mean 100 was used for each hierarchical standard deviation (σ_j). In the models where a parameter (a, c, or d) was common to all farms, a normal prior with mean 0 and standard deviation 10 was used for the parameter. Finally, normal priors with mean 0 and standard deviation 10 were used for the components of the piecewise force of infection (i.e., the b_ks).

Samples from the joint posterior density were generated using an adaptive Metropolis scheme (*19*), modified so that the scaling factor was tuned during burn-in to ensure an acceptance rate of between 20% and 40% for more efficient sampling of the target distribution (*20*). Two chains of 2,000,000 iterations were run, with the first 1,000,000 iterations discarded to allow burn-in of the chains. Each chain was subsequently thinned by taking every 100th iteration. The adaptive Metropolis scheme was implemented in Matlab (version R2020b; The Mathworks Inc.). Convergence of the scheme was assessed visually and by the Gelman-Rubin statistic in the coda package (*21*) in R (version 4.0.5) (*11*).

Different models for the force of infection, vaccine efficacy and effect of other control measures were compared using the deviance information criterion (DIC) (*22*), with a model having a smaller DIC preferred to one with a larger DIC.

Results

Field Study 2: Follow-up Study

Most lambs (n = 120; 96.0%) were kept indoors all the time while adults were kept indoors at night and outdoors during the day. Only 9.1% of the animals (n = 34; from 11 different farms) had ticks on the day of sampling. The number of seropositive animals was 181 (48.3%) for CCHFV-Gc IgG, 74 (19.7%) for CCHFV-NP IgG.

Field study 1: Cross-sectional study

Animal level

Age distribution of sheep sampled as part of the cross-sectional study conducted in October 2017 were analyzed (Appendix Figure 1).

Farm level

Farm profiles related to farm characteristics and management practices were identified. Following MCA treatment, dimension one separated farms by geographic location (province) and production type (explaining 27.2% of the variance), whereas dimension two separated farms by management practices and explained 11.1% of the variance. Three typologies (clusters) were identified following HCA. The contribution of each variable is presented in (Appendix Table 3).

References

1. Mertens M, Schuster I, Sas MA, Vatansever Z, Hubalek Z, Güven E, et al. Crimean-Congo hemorrhagic fever virus in Bulgaria and Turkey. Vector Borne Zoonotic Dis. 2016;16:619–23. PubMed https://doi.org/10.1089/vbz.2016.1944

2. Panayotova E, Papa A, Trifonova I, Christova I. Crimean-Congo hemorrhagic fever virus lineages Europe 1 and Europe 2 in Bulgarian ticks. Ticks Tick Borne Dis. 2016;7:1024–8. PubMed https://doi.org/10.1016/j.ttbdis.2016.05.010

3. Barthel R, Mohareb E, Younan R, Gladnishka T, Kalvatchev N, Moemen A, et al. Seroprevalance of Crimean-Congo haemorrhagic fever in Bulgarian livestock. Biotechnol Biotechnol Equip. 2014;28:540–2. PubMed https://doi.org/10.1080/13102818.2014.931685

4. Christova I, Gladnishka T, Taseva E, Kalvatchev N, Tsergouli K, Papa A. Seroprevalence of Crimean-Congo hemorrhagic fever virus, Bulgaria. Emerg Infect Dis. 2013;19:177–9. PubMed https://doi.org/10.3201/eid1901.120299

5. Otte MJ, Gumm ID. Intra-cluster correlation coefficients of 20 infections calculated from the results of cluster-sample surveys. Prev Vet Med. 1997;31:147–50. PubMed https://doi.org/10.1016/S0167-5877(96)01108-7

6. Deem SL, Perry BD, Katende JM, McDermott JJ, Mahan SM, Maloo SH, et al. Variations in prevalence rates of tick-borne diseases in Zebu cattle by agroecological zone: implication for East

Coast fever immunization. Prev Vet Med. 1993;16:171–87. https://doi.org/10.1016/0167-5877(93)90064-Z

7. Belij-Rammerstorfer S, Limon G, Maze EA, Hannant K, Hughes E, Tchakarova SR, et al. Development of anti–Crimean-Congo hemorrhagic fever virus Gc and NP-specific ELISA for detection of antibodies in domestic animal sera. Front Vet Sci. 2022;9:913046. PubMed https://doi.org/10.3389/fvets.2022.913046

8. Estrada-Peña A, J. de la Fuente J. Species interactions in occurrence data for a community of tick-transmitted pathogens. Sci Data. 2016;3:160056. PubMed https://doi.org/10.1038/sdata.2016.56

9. Hijmans RJ, Guarino L, Mathur P. DIVA-GIS. 2012 [cited 2019 Feb 15]. https://diva-gis.org

10. Pfeiffer DU, Robinson TP, Stevenson M, Stevens KB, Rogers DJ, Clements ACA. Local estimates of spatial clustering. In: Spatial analysis in epidemiology. New York: Oxford University Press; 2008.

11. R Core Team. R: a language and environment for statistical computing. R Foundation for Statistical Computing. 2021 [cited 2024 Nov 1]. http://www.R-project.org

12. Fox J, Weisberg S. An R companion to applied regression, third edition. Thousand Oaks, CA, USA: Sage Publications; 2018.

13. Lê S, Josse J, Husson F. FactoMineR: an R package for multivariate analysis. J Stat Softw. 2008;25:1–18. https://doi.org/10.18637/jss.v025.i01

14. Bates D, Mächler M, Bolker B, Walker S. Fitting linear mixed-effects models using lme4. J Stat Softw. 2015;67:1–48. https://doi.org/10.18637/jss.v067.i01

15. Zeileis A, Hothorn T. Diagnostic checking in regression relationships. R News. 2002;2:7–10.

16. Environmental Systems Research Institute, Inc. ArcMap version 10.8.1 [cited 2024 Oct 1]. https://desktop.arcgis.com/en/arcmap/index.html

17. Buttigieg KR, Dowall SD, Findlay-Wilson S, Miloszewska A, Rayner E, Hewson R, et al. A novel vaccine against Crimean-Congo haemorrhagic fever protects 100% of animals against lethal challenge in a mouse model. PLoS One. 2014;9:e91516. PubMed https://doi.org/10.1371/journal.pone.0091516

18. Halloran ME, Longini IM, Struchiner CJ. Design and analysis of vaccine studies. New York: Springer Nature; 2010.

19. Haario H, Saksman E, Tamminen J. An adaptive Metropolis algorithm. Bernoulli. 2001;7:223–42. https://doi.org/10.2307/3318737

20. Andrieu C, Thoms J. A tutorial on adaptive MCMC. Stat Comput. 2008;18:343–73. https://doi.org/10.1007/s11222-008-9110-y

21. Plummer M, Best N, Cowles K, Vines K. CODA: convergence diagnosis and output analysis for MCMC. R News. 2006;6:7–11.

22. Spiegelhalter DJ, Best NG, Carlin BP, van der Linde A. Bayesian measures of model complexity and fit. J R Stat Soc Series B Stat Methodol. 2002;64:583–639. https://doi.org/10.1111/1467-9868.00353

Appendix Table 1. Data management and variable reclassification used for analysis

Questionnaire				
Variable	Possible answer		Data recategorization for analysis	
Production type	Dairy, meat, wool	Could be >1 option	Dairy only, meat only, mixed	NA
Place where ewes originated	Same farm, neighboring farms, middlemen, livestock markets, or other (specify)	Could be >1 option	Own replacements only or a combination of own replacements and elsewhere	NA
Place where rams originated	Same farm, neighboring farms, middlemen, livestock markets, or other (specify)	Could be >1 option	Own replacements only, always from other farms, or a combination of own replacements and elsewhere	NA
Place where lambs were kept until weaning	Outdoors all the time, indoors all the time, part of the time outdoors and part of the time indoors	NA	Indoors all the time, outdoors during the day and indoors at night	NA
Number of ewes (>1 y old) on the day of the visit	Continuous variables		≤75 or >75	Skewed to the right, median number used as cutoff
Number of rams (>1 y old) on the day of the visit			≤2 or >2	
Number of lambs (≤1 y old) on the day of the visit			≤11 or >11	
Other livestock species in the farm. *If yes*, which species: cattle, buffalo, goats, pigs, other (specify)	Yes or no		Keep cattle, no or yes; keep goats, no or yes	NA
Tick control for animals in the farm. If yes, type of tick control	Yes, no; spraying, dipping, salt with repellent	NA	No; dipping, spraying	NA
Deworming, common practice to deworm sheep in this farm. *If yes*, product used.	Yes, no, open question	NA	No; deworm with ivermectin; deworm with a product different to ivermectin	NA

NA, not applicable.

Appendix Table 2. Contribution of each characteristic considered for the multiple correspondence analysis

Characteristic	Contribution	
	Dimension 1	Dimension 2
Province	0.804	0.033
Production type	0.733	0.442
Ewes from	0.005	0.180
Rams from	0.329	0.400
Place where lambs are kept until weaning	0.700	0.047
Number of ewes	0.512	0.067
Number of rams	0.391	0.098
Number of lambs	0.533	0.073
Keep cattle	0.338	0.001
Keep goats	0.062	0.112
Tick control (type)	0.069	0.414
Deworming (type)	0.141	0.026

Appendix Table 3. Characteristics of farms belonging to typologies (clusters) identified by hierarchical cluster analysis (HCA) including the 120 farms that were part of the cross-sectional study.

Characteristic	Typology 1, no. (%)	Typology 2, no. (%)	Typology 3, no. (%)
No. farms	40	26	53
Province			
Burgas	1 (1.7)	7 (11.7)	52 (86.7)
Kardhzali	39 (65.0)	19 (31.7)	2 (3.3)
Production type			
Dairy only	2 (3.2)	9 (14.3)	52 (82.5)
Meat only	5 (21.7)	10 (73.9)	1 (4.3)
Mixed	33 (97.1)	0	1 (2.9)
Ewes from			
Own replacements only	40 (35.1)	22 (19.3)	52 (45.6)
Combination of own replacements and elsewhere	0	4 (66.7)	2 (33.3)
Rams from			
Own replacements only	39 (65.0)	8 (13.3)	13 (21.7)
Always from other farms	0	0	16 (100)
Combination of own replacements and elsewhere	0	18 (41.9)	25 (58.1)
Place where lambs are kept until weaning			
Indoors all the time	1 (1.9)	7 (13.2)	45 (84.9)
Outdoors during the day and indoors at night	39 (58.2)	19 (28.4)	9 (13.4)
No. ewes			
≤75	29 (47.5)	23 (37.7)	9 (14.8)
>75	11 (18.6)	3 (5.1)	45 (76.3)
No. rams			
≤2	26 (40.6)	25 (39.1)	13 (20.3)
>2	14 (25.0)	1 (1.8)	41 (73.2)
No. lambs			
≤11	30 (48.4)	24 (38.7)	8 (12.8)
>11	10 (17.2)	2 (3.4)	46 (79.3)
Keep cattle			
No	20 (24.7)	10 (12.3)	51 (63.0)
Yes	20 (51.3)	16 (41.0)	3 (7.7)
Keep goats			
No	31 (33.7)	26 (28.3)	35 (38.0)
Yes	9 (32.1)	0	19 (67.9)
Tick control			
No	0	10 (71.4)	4 (28.6)
Dipping	0	0	5 (100)
Spraying	40 (39.6)	16 (15.8)	45 (44.6)
Deworm, type			
No	1 (20.0)	3 (60.0)	1 (20.0)
Deworm with ivermectin	13 (27.1)	6 (12.5)	29 (60.4)
Deworm with product different from ivermectin	26 (38.8)	17 (25.4)	24 (35.8)

Appendix Table 4. Univariate analysis between animal age and serostatus for all farms sampled in the follow-up study (n = 375)*

	Seropositive for CCHFV GP Gc IgG			Seropositive for CCHFV NP IgG		
Age group†	No. positive (%)	OR (95% CI)	p value	No. positive (%)	OR (95% CI)	p value
0–12 mo., lambs, n = 125	63 (50.4)	Referent		24 (19.2)	Referent	
13–36 mo., young adults, n = 127	61 (48.0)	0.86 (0.46–1.59)	0.624	26 (20.5)	1.16 (0.38–1.95)	0.709
>36 mo., adults, n = 123	57 (46.3)	0.78 (0.42–1.45)	0.426	24 (19.5)	1.01 (0.21–1.81)	0.977

*All models include farm as a random effect. Data were collected in March 2018. CCHFV, Crimean-Congo hemorrhagic fever virus; GP, glycoprotein; NP, nucleoprotein; OR, odds ratio.
†Categorized according to follow-up study design.

Appendix Table 5. Univariate analysis between animal age and serostatus considering only farms that were visited in both field studies (n = 14) and sheep of the same age (3–24 mo.) (n = 198)*

	Seropositive for CCHFV GP Gc IgG			Seropositive for CCHFV NP IgG		
Characteristic	No. positive (%)	OR (95% CI)	p value	No. positive (%)	OR (95% CI)	p value
Study						
Follow up, March 2018, n = 58	22 (37.9)	Referent		5 (8.6)	Referent	
Cross-sectional, Oct 2017, n = 140	55 (39.3)	1.41 (0.70–2.93)	0.335	75 (53.6)	15.37 (5.91–49.46)	<0.001
Age category						
13–24 mo., young adults, n = 115	41 (35.7)	Referent		38 (33.0)	Referent	
3–12 mo., lambs, n = 83	36 (43.4)	1.51 (0.79–2.93)	0.214	42 (50.6)	2.18 (1.20–4.06)	0.012

*All models include farm as a random effect. Data were collected in March 2018. CCHFV, Crimean-Congo hemorrhagic fever virus; GP, glycoprotein; NP, nucleoprotein; OR, odds ratio.

Appendix Table 6. Weighted seroprevalence stratified by province*

	Weighted seroprevalence, % (95% CI)	
Province	CCHFV GP Gc IgG	CCHFV NP IgG
Overall	40.0 (36.8–43.0)	38.5 (35.3–42.0)
Burgas	42.2 (37.9–47.0)	45.2 (40.9–50.0)
Kardzhali	34.4 (30.7–38.0)	21.7 (18.3–25.0)

*CCHFV, Crimean-Congo hemorrhagic fever virus; GP, glycoprotein; NP, nucleoprotein.

Appendix Table 7. Univariate analysis between animal age and serostatus (n = 1200 sheep)*

	Seropositive for CCHFV NP IgG		
Characteristic	No. positive (%)	OR (95% CI)	p value
Province			
Kardzhali, n = 600	140 (33.90)	Referent	
Burgas, n = 600	273 (66.10)	3.20 (2.12–4.93)	<0.001
Sex			
F, n = 1,163	403 (97.58)	Referent	
M, n = 37	10 (2.42)	0.74 (0.07–1.57)	0.489
Age category			
3–12 mo., lambs, n = 596	199 (48.18)	Referent	
13–24 mo., young adults, n = 604	214 (51.82)	1.08 (0.82–1.34)	0.548
Breed†			
Mixed breed, n = 743	190 (46.12)	Referent	
Meat breeds, n = 20	217 (52.67)	0.70 (0.01–2.52)	0.705
Dairy breeds, n = 436	5 (1.21)	2.11 (1.67–2-57)	0.001
Ticks present during sampling			
No, n = 1,149	391 (94.67)	Referent	
Yes, n = 51	22 (5.33)	1.37 (0.69–2.05)	0.364

*All models include farm as a random effect. Data were collected in October 2017. CCHFV, Crimean-Congo hemorrhagic fever virus; NP, nucleoprotein; OR, odds ratio.
†Dairy breeds included: Awasi, Lacune, Pleven black and Splotch-faced Marishka, and Synthetic Milk; meat breeds included Karnobatska and Starop ysigay; and mixed breed refers to cross breeds.

Appendix Table 8. Results for multivariable analysis for identification of risk factors for CCHFV seropositivity at farm level using Poisson regression models*

	Seropositive for CCHFV NP IgG	
Characteristic	aPR (95% CI)	p value
Farm typology		
Typology 2	Referent	
Typology 1	0.80 (0.66–0.97)	0.022
Typology 3	1.24 (1.05–1.48)	0.014
Land cover		
Shrub	Referent	
Cultivated	1.47 (1.22–1.77)	<0.001
Arboreal	1.71 (1.29–2.24)	0.0001

*Number of animals sampled in the farm was used as offset and number of seropositive animals was used as outcome. Data were collected in October 2017. aPR, adjusted prevalence ratio; CCHFV, Crimean-Congo hemorrhagic fever virus; NP, nucleoprotein.

Appendix Table 9. Characteristics of lambs included in the vaccine intervention study*

Characteristics	Placebo	Vaccine
Sex		
F	166 (50.3%)	164 (49.7%)
M	154 (49.7%)	156 (50.3%)
Breed		
Asaf	22 (50.0%)	22 (50.0%)
Avasi	8 (42.1%)	11 (57.9%)
Lacume	19 (47.5%)	21 (52.5%)
Mixed	95 (51.1%)	91 (48.9%)
Synthetic Milk	176 (50.1%)	175 (49.9%)
Median age, d (range)	59 (27–88)	59 (27–88)

*Values are no. (%) except as indicated. Vaccine intervention study is field study 3.

Appendix Table 10. Deviance information criterion (DIC) comparing models for FOI and vaccine efficacy*

	Model	
Constant FOI, common V_E	FOI varies with sampling period, common V_E	FOI varies with sampling period, V_E varies among farms
1960.5	1902.1	1883.0

*A model with a lower DIC is preferred to one with higher DIC.

Appendix Table 11. Deviance information criterion (DIC) comparing models for the effect of different control measures*

Control measures†				
Deworming	Tick control		Spraying	
Common	Common	Varied	Common	Varied
1883.9	1884.6	1868.5	1879.3	1867.6

*A model with a lower DIC is preferred to one with higher DIC.

Appendix Table 12. Distribution of potential risk factors for CCHFV seropositive farmers according to univariate analysis (n = 44 farmers) using mixed-effect models, including farm as a random effect*

			Univariate analysis	
Characteristic	No. seronegative (%)	No. seropositive (%)	OR	p value
Province in Bulgaria				
Kardzhali	20 (100)	0	NA	
Burgas	21(87.5)	3 (12.5)	NA	NA
Sex				
F	18 (94.7)	1 (5.2)	Referent	
M	23 (92.0)	2 (8.0)	1.56	0.72
Age, y				
<u><</u>51	21 (95.4)	1 (4.5)	Referent	
>51	20 (90.9)	2 (9.1)	2.10	0.56
Assistance during animals' parity				
No	7 (87.5)	1 (12.5)	Referent	
Yes	34 (94.4)	2 (5.6)	0.41	0.49

Characteristic	No. seronegative (%)	No. seropositive (%)	Univariate analysis	
			OR	p value
Assistance when slaughtering animals in the farm				
No	11 (100)	0	NA	
Yes	30 (90.9)	3 (9.1)	NA	
Assistance when butchering meat				
No	7 (100)	0	NA	
Yes	34 (91.9)	3 (8.1)	NA	
Milk animals manually				
No	5 (83.3)	1 (16.7)	Referent	
Yes	36 (94.7)	2 (5.3)	0.27	0.33
Have been bitten by ticks				
No	26 (96.3)	1 (3.7)	Referent	
Yes	15 (88.2)	2 (11.8)	3.5	0.33
Have been vaccinated against CCHFV				
No	40 (95.2)	2 (4.8)	Referent	
Yes	1 (50.0)	1 (50.0)	20.0	0.06

*CCHFV, Crimean-Congo hemorrhagic fever virus; NA, not applicable; OR, odds ratio.

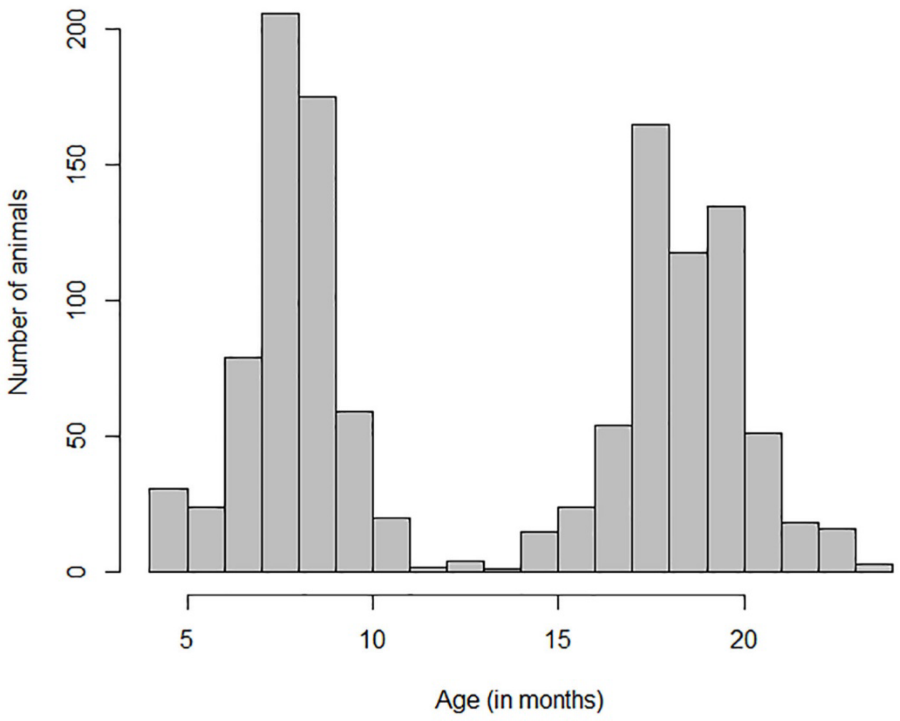

Appendix Figure 1. Age distribution of sheep sampled as part of the cross-sectional study conducted in October 2017.

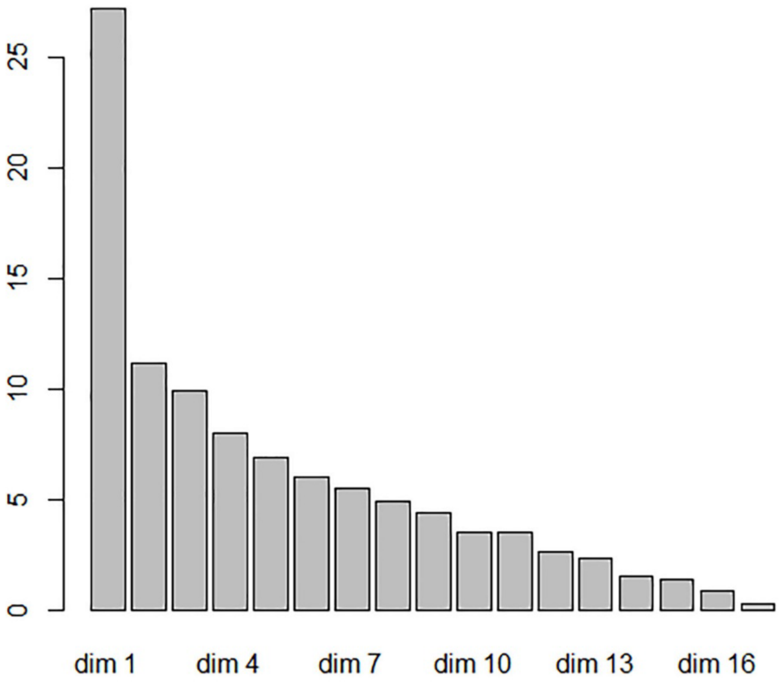

Appendix Figure 2. Eigenvalues in each dimension. dim, dimension.

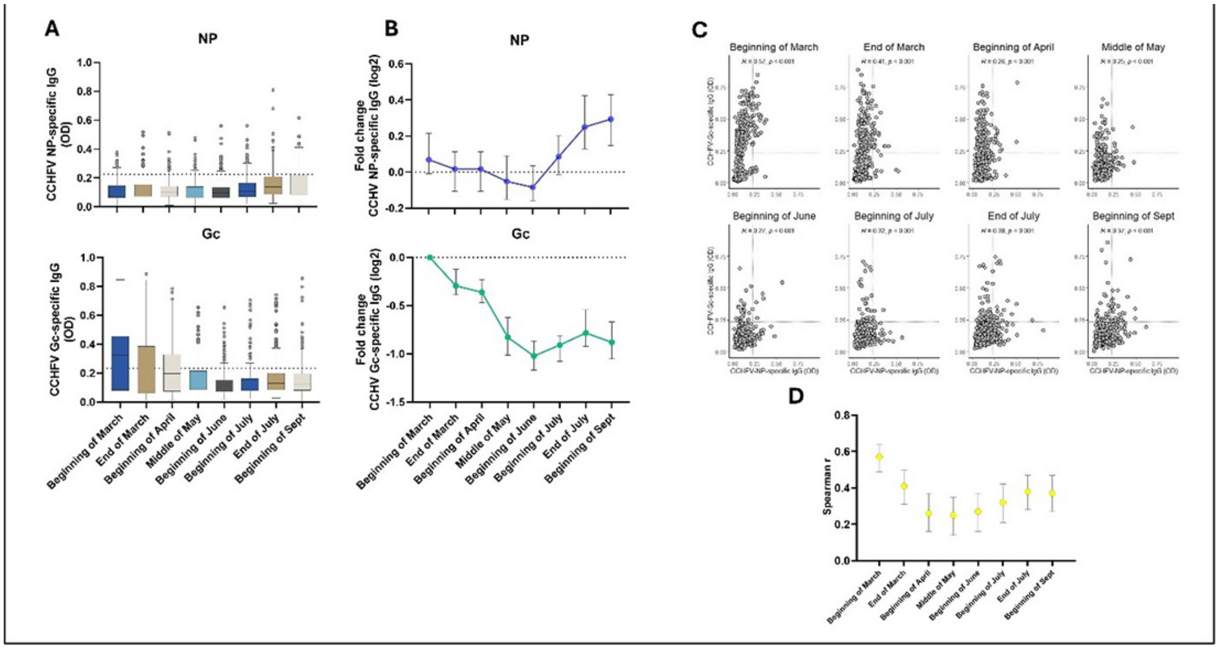

Appendix Figure 3 . Profiles of CCHFV NP and Gc IgG seroreactivity in lambs naturally exposed to the virus. A) Boxplots showing OD values for CCHFV NP (top) and CCHFV-Gc IgG (bottom) IgG measured by ELISA across 8 timepoints during March–September, 2017. Each box represents the interquartile range (IQR), the line indicates the median, and whiskers denote 1.5 × IQR. Dotted lines mark the

seropositivity cutoffs (0.225 for NP, 0.234 for Gc). B) Median $\log_2$-fold change in CCHFV-NP (top) and CCHFV-Gc (bottom) IgG OD relative to day 0 (beginning of March), with 95% CI. C) Scatter plots showing the correlation between CCHFV-NP and CCHFV-Gc IgG ODs at each timepoint. Each dot represents a sample; dotted lines represent positivity cutoffs. Spearman correlation coefficients (r) with 95% CI and associated p values are shown in each panel. D) Spearman correlation coefficients (r ± 95% CI) over time between CCHFV-NP- and CCHFV-Gc IgG. CCHFV, Crimean-Congo hemorrhagic fever virus, Gc, glycoprotein Gc, NP, nucleoprotein, OD, optical density.

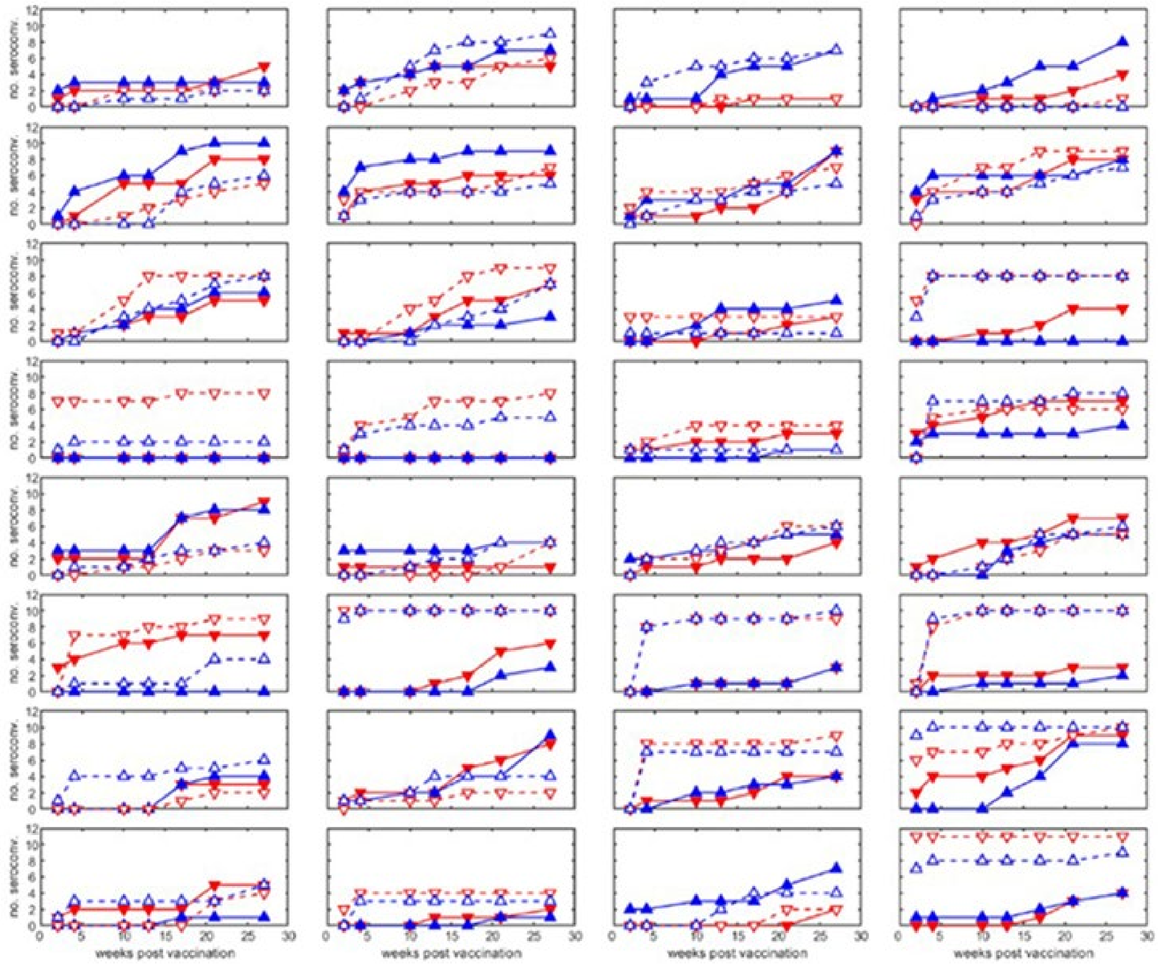

Appendix Figure 4 . Cumulative number of lambs seroconverting to Crimean Congo hemorrhagic fever virus IgG on 32 sheep farms in Bulgaria. Each plot shows the number of unvaccinated (red, down-triangles) and vaccinated (blue, up-triangles) lambs from each of the 32 farms seroconverting according to NP ELISA (solid line, filled triangles) or Gc ELISA (dashed lines, open triangles) results.

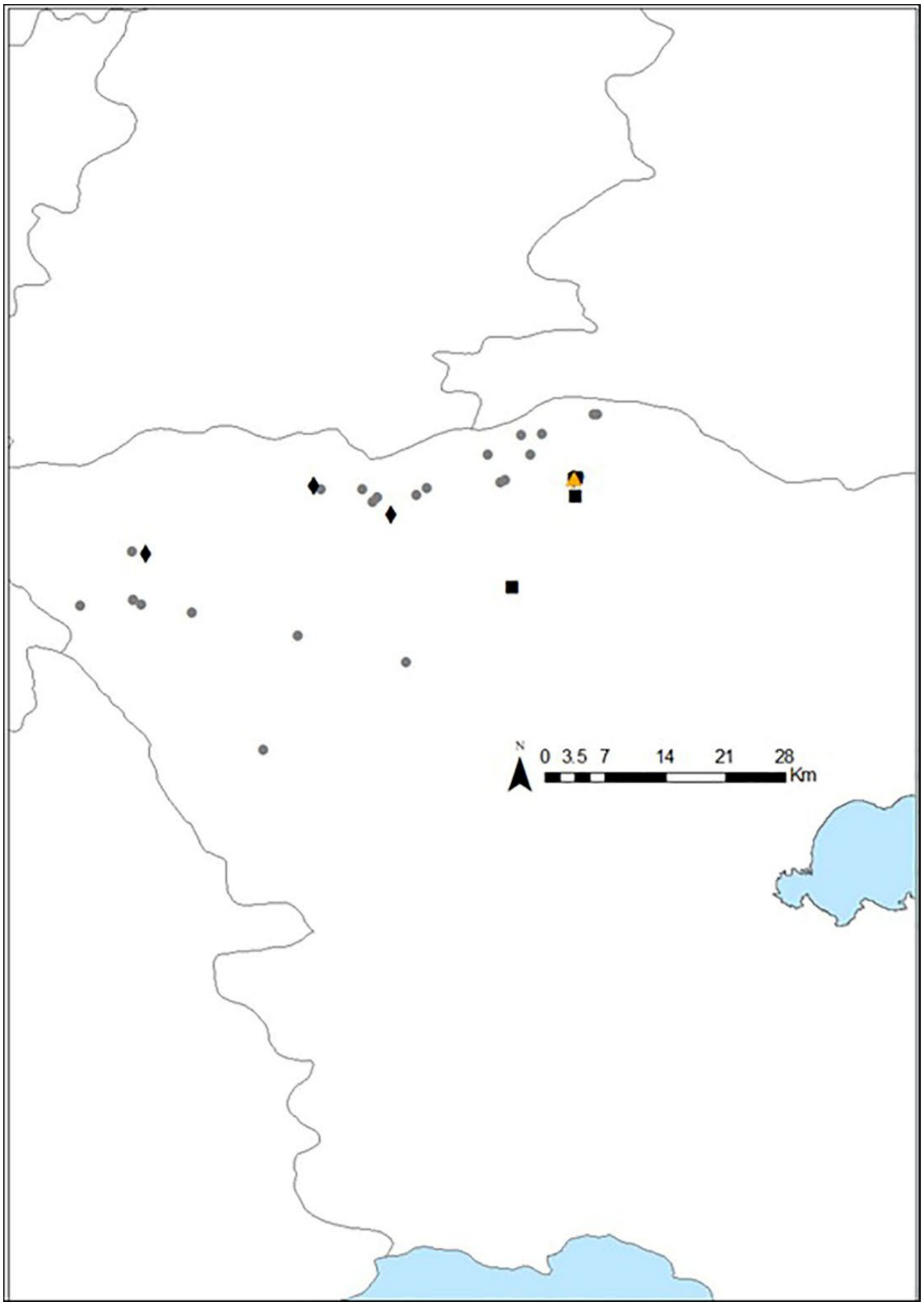

Appendix Figure 5. Geographic location of farms with the highest force of infection (FOI) on the basis of the presence of CCHFV-Gc IgG (black square), CCHFV-NP IgG (black diamond), and those with high FOI to both antigens (orange triangle).
